# Quantification of floating riverine macro-debris transport using an image processing approach

**DOI:** 10.1038/s41598-020-59201-1

**Published:** 2020-02-10

**Authors:** Tomoya Kataoka, Yasuo Nihei

**Affiliations:** Department of Civil Engineering, Faculty of Science and Technology, Tokyo University of Science, Chiba, 278-8510 Japan

**Keywords:** Civil engineering, Environmental impact, Computational science

## Abstract

A new algorithm has been developed to quantify floating macro-debris transport on river surfaces that consists of three fundamental techniques: (1) generating a difference image of the colour difference between the debris and surrounding water in the CIELuv colour space, (2) detecting the debris pixels from the difference image, and (3) calculating the debris area flux via the template matching method. Debris pixels were accurately detected from the images taken of the laboratory channel and river water surfaces and were consistent with those detected by visual observation. The area fluxes were statistically significantly correlated with the mass fluxes measured through debris collection. The mass fluxes calculated by multiplying the area fluxes with the debris mass per unit area (M/A) were significantly related to the flood rising stage flow rates and agreed with the mass fluxes measured through debris collection. In our algorithm, plastic mass fluxes can be estimated via calibration using the mass percentage of plastics to the total debris in target rivers. Quantifying riverine macro-plastic transport is essential to formulating countermeasures, mitigating adverse plastic pollution impacts and understanding global-scale riverine macro-plastic transport.

## Introduction

Quantifying the transport of macro-debris floating on the world’s rivers, which are major sources of ocean debris, is essential in formulating countermeasures to mitigate the adverse impacts of land-based loads. In particular, the adverse impacts on aquatic ecosystems of plastics containing toxic chemicals (e.g., persistent organic pollutants (POPs))^1–4^ are recognized as a serious concern in the global aquatic environment^5–8^. Many of the plastics in the oceans originate from land^9^, and thereafter, macro-plastics (>25 mm in diameter)^10^ are evenly broken down into smaller plastic fragments known as meso- (5−25 mm in diameter) and micro-plastics (<5 mm in diameter)^10^ due to photo- and thermo-oxidative degradation^6^. Micro-plastics are rarely removed from the aquatic environment when released and are thus gradually transported far away due to ocean currents. Hence, to formulate countermeasures against oceanic plastic pollution, the macro-plastics in rivers must be efficiently captured before being fragmented into smaller pieces and/or being released to the oceans. In particular, because most macro-plastics float on the surface, it is important to understand how floating macro-debris is transported via rivers and then released into the oceans.

Recently, a few studies have attempted to estimate the plastic waste emissions from land^9,11^. Jambeck *et al*.^9^, for instance, estimated that 4.8 to 12.7 million tonnes of mismanaged plastic waste could be entering the oceans from 192 coastal countries in 2010 by considering the waste management level in each country and the coastal population. In their estimate, 0.02 to 0.06 million MT of plastic waste can enter the Pacific Ocean from Japan. Moreover, Lebreton *et al*.^11^ estimated that between 1.15 and 2.41 million tonnes of plastic waste could be entering the oceans from rivers every year by applying a global model of plastic inputs from rivers based on waste management, population density and hydrological information. These two estimates of land-based plastic waste were based on data on the mean waste generation in kilograms per inhabitant and per day in each country, although waste generation differs spatially among the various regions of each country because of different land uses. These estimates, therefore, need to be verified in each country using more detailed data.

As a suitable method to verify these estimates, we focus on quantifying floating macro-debris based on field surveys. Recently, several studies have investigated micro-plastic contamination in rivers around the world^12–19^, while there have only been a few studies on collecting, monitoring and quantifying floating macro-debris based on field surveys^20–23^. Nihei *et al*.^20^ collected floating macro-debris using a net (2.5 cm mesh) at the Noda Bridge across the Edo River, Japan, and reported that the proportion of anthropogenic debris to the total debris was approximately 6% by weight. In addition, Gasperi *et al*.^21^ reported that plastic debris represented between 0.8% and 5.1% of the total macro-debris by weight and annually intercepted between 22 and 36 tons of floating plastic debris through the use of floating debris-retention booms. Recently, a tablet computer application has been developed that harmonizes visual observations from debris monitoring to systematically gather comparable floating macro-debris data involving many activities and institutes in various countries^23^. The application is useful to efficiently record characteristics of the debris (e.g., size, item, and number) on river surfaces. These previous methods can gather reliable data on the quantity and characteristics of floating macro-debris, although they are labour intensive and costly. In addition, these methods experience difficulties in sequentially observing the temporal fluctuations in the debris quantity.

Video monitoring of river surfaces could be an effective approach to safely and efficiently quantify floating macro-debris transport. However, there have been very few studies on remote monitoring of riverine debris except for our research^24,25^, although the macro-debris in marine environments has already been quantified using webcams^26–28^, aerial photography^29,30–35^, satellite imagery^36^, and light detection and ranging (LIDAR) technology^37^. It is difficult to apply aerial photography, satellite imagery, and LIDAR technology because monitoring with a high temporal resolution is needed in rivers. In particular, because a large quantity of floating macro-debris regularly flows downstream during floods due to heavy rainfall^38^, quantifying the floating macro-debris that is transported under flood conditions is crucial to assess the land-based loads of riverine macro-debris in the oceans. Traditionally, the fluxes of pollutants in rivers have been related to flow rates (i.e., the water volume per unit of time)^11,39,40^. If a relationship between the floating macro-debris fluxes and flow rates can be established through video monitoring, we can not only verify the estimates of plastic waste input to the oceans but also manage the land-based plastic waste emissions. Thus far, we have been attempting to develop a technique for quantifying the debris transport on river surfaces using video data^24,25^. Our previous studies exhibit a weakness in the detection of debris pixels. We experienced difficulty in extracting the debris pixels of large items and conglomerations because an edge detection algorithm was implemented. Thus, the detection accuracy is low because the pixels around the edge of the debris are detected, while the pixels around its centre are unable to be detected using this algorithm.

Here, we develop a new algorithm for quantifying floating riverine macro-debris transport, which consists of three fundamental techniques. First, an image of the colour difference between the debris and water surface (difference image) is obtained. Next, debris pixels are detected by binarizing the difference image using a constant threshold value. Finally, the area of the floating macro-debris that is passively transported per unit time (area flux) is calculated by applying the template matching method. To verify the performance of our algorithm, both laboratory and river experiments were conducted. In the laboratory experiment, natural and anthropogenic items floating along an open channel were filmed perpendicularly using a digital video camera installed at the top of the channel to verify the detection performance for the items floating on the water surface. In the river experiment, floating macro-debris in the Edo River was collected using a net, and the river surface on the upstream side of the net was recorded using a camera. In both experiments, the area covered by debris (covered area) was evaluated by counting the pixels identified as debris to validate the detection accuracy. Furthermore, we examine the applicability of the mass flux estimation method based on the area flux. This paper presents a basis for remotely assessing the mass flux using digital video cameras in rivers and can be useful in formulating waste management guidelines in different countries.

## Results

### Verification of the performance of our technique in the laboratory experiment

**Figure 1:**
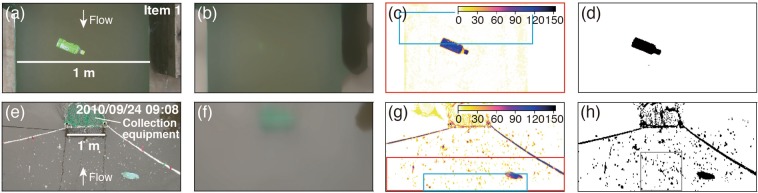
Images of the image processing flow steps of our algorithm. Panels (a–h) show the laboratory experiment and river monitoring, respectively. Panels (a) and (e) are the original images, (b) and (f) are the smoothed images, (c) and (g) are the difference images, and (d) and (h) are the binary images. The colour scale of the difference images is shown at the upper-right of panels (c) and (g). The red and blue boxes in panels (c) and (g) are the analysis and template planes shown in Fig. 6, respectively. The grey box in panel (h) is the calculated covered area.

To investigate the detection performance of floating macro-debris on the water surface, the covered area and transport velocity of the twenty items (Supplementary Fig. S1 and Table S1) were verified in the laboratory experiment. The twenty items were successfully detected (Supplementary Fig. S2) by calculating the colour difference in the CIELuv colour space^41^ (see Supplementary Notes) between each original frame extracted from the video (Fig. 1a) and its smoothed frame (Fig. 1b). The colour difference of the debris pixels was significantly high (Fig. 1c), and the debris pixels were extracted by selecting a colour difference of 10 as the threshold value (Fig. 1d).

**Figure 2:**
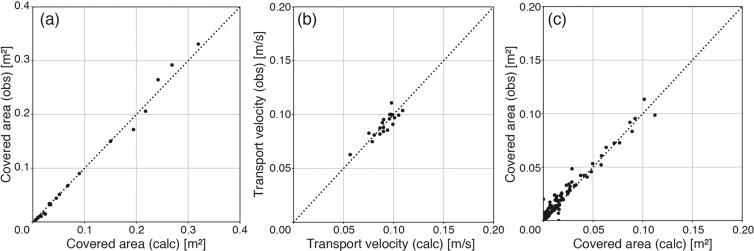
Scatterplots of the covered area (**a**,**c**) and transport velocity (**b**) calculated by our algorithm versus those measured by the observers. Panels (a) and (c) show the comparison of the covered areas obtained in the laboratory and river experiments, respectively. Panel (b) shows the comparison between the transport velocities measured by a stopwatch and calculated via image processing. The line *y* = *x* is added to each panel.

The covered areas of the twenty items were calculated by analysing the videos, and consequently, the calculated covered areas agreed well with their true projected areas measured by observers (*n* = 20, *r* = 0.996, and *p* < 0.001; Fig. 2a). In addition, the mean transport velocity calculated from all frames of each video was also consistent with that measured using a stopwatch (*n* = 20, *r* = 0.879, and *p* < 0.001; Fig. 2b). These results demonstrate that our algorithm enables us to measure the area flux using video data.

### Verification of the applicability of our technique in the river experiment

The applicability of our algorithm was verified by processing 29 videos that were perpendicularly recorded using a digital video camera at the Noda Bridge across the Edo River during the flooding events caused by the two typhoons that occurred in 2010 (see Supplementary Notes). The verification was conducted via two approaches. First, the covered areas were compared with those calculated by extracting the debris pixels from each frame by visual observation. For comparison, both covered areas in the grey boxed area shown in Fig. 1h were calculated from multiple frames derived from four videos (videos 1, 2, 3 and 6; Supplementary Table S2) at one-second intervals. The covered area calculated via image processing was very consistent with that calculated via visual observation (*n* = 372, *r* = 0.983, and *p* < 0.001; Fig. 2c), indicating that our algorithm can be successfully applied to detect debris pixels.

**Figure 3:**
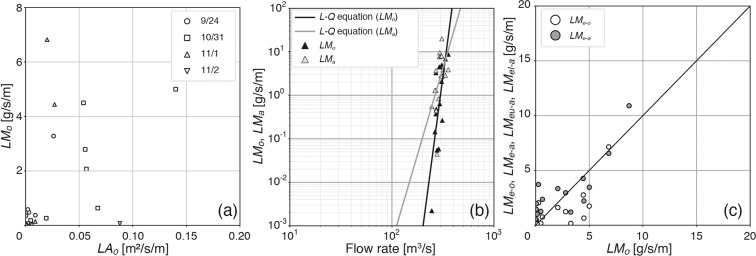
Scatterplots of the area flux (*LA*_*o*_) calculated with our algorithm versus the mass flux (*LM*_*o*_) measured using the collection equipment (**a**), the relationship between *LM*_*o*_ and mass flux (*LM*_*a*_) computed from *LA*_*o*_ with the flow rate (**b**), and the correlation between the mass fluxes (*LM*_*e-o*_, *LM*_*e-a*_) estimated by substituting the flow rate for the *L*-*Q* equations (**c**). In panel (a), the symbols are based on the collection date of floating macro-debris. The black and grey solid lines in panel (b) are the *L*-*Q* equations for *LM*_*o*_ and *LM*_*a*_, respectively. The symbols are shown in the box of each panel. The line *y* = *x* is added to panel (c).

Next, the relationship between the area and mass fluxes was investigated by comparing both fluxes based on the 29 observations (Supplementary Table S2). The area flux was calculated by dividing the summation of the surface area of the debris that is transported at the analysis frame rate (transport areas) across the width of the collection equipment (1 m; see Fig. 1e) by the filming time, which is referred to as *LA*_*o*_. In addition, the mass flux was measured by dividing the total mass of the debris captured by the collection equipment by the collection duration, which is referred to as *LM*_*o*_. Thus, both fluxes were defined as fluxes per unit width (m^2^/s/m and g/s/m, respectively). Comparing *LA*_*o*_ and *LM*_*o*_ based on the 29 observations (Fig. 3a), *LA*_*o*_ was significantly correlated with *LM*_*o*_ (*n* = 29, *r* = 0.447, and *p* < 0.05; Fig. 3a). The significant relationship makes it possible to estimate the mass flux from the area flux. Nevertheless, as expected, *LM*_*o*_ was much more variable than *LA*_*o*_. The large variance was caused by the mass per unit area of the debris (i.e., the ratio of *LM*_*o*_ to *LA*_*o*_; hereinafter referred to as M/A). M/A depends on the debris composition and different volumes, shapes and weight densities of each debris item. The mean M/A was 0.14 ± 0.05 kg/m^2^, and its relative error was 35% (Supplementary Table S2). Notably, the uncertainty was defined as the standard error of the mean (SEM), and thus the 95% confidence interval was calculated by multiplying the SEM by the *t* value for a 5% two-tailed probability with 28 degrees of freedom (*t*_0.05_ = 2.048).

### Estimation of the mass flux from the area flux

The significant relationship between *LA*_*o*_ and *LM*_*o*_ can be used to estimate mass fluxes without collecting debris. The simplest estimation of the mass flux is to multiply the area flux by the mean M/A (0.14 kg/m^2^), which is hereafter referred to as *LM*_*a*_. The root mean square error (RMSE) between *LM*_*o*_ and *LM*_*a*_ was 4 g/s/m. Here, to demonstrate the significance of the area flux, *LM*_*o*_ was compared with *LM*_*a*_ relative to the flow rate.

As with the traditional modelling of suspended matter^40^, *LM*_*o*_ and *LM*_*a*_ can be modelled by the following equation, which is referred to as the *L-Q* equation.

1 $$L=a{Q}^{b}$$

where $L$ and $Q$ are the mass flux and flow rate, respectively. Coefficients $a$ and $b$ are determined by the least squares method using the mass fluxes (*LM*_*o*_ and *LM*_*a*_) and flow rate. Coefficient $b$ is the most important parameter for determining the transport characteristics of the floating macro-debris in a river. Additionally, if the coefficients can be correctly determined, the mass fluxes can be estimated from the flow rate without river video monitoring.

First, both *LM*_*o*_ and *LM*_*a*_ at the rising stage, in which the difference in the water level per unit time (hereafter referred to as the water level rate) is greater than 0, were regressed using Eq. (1) (Fig. 3b), as summarised in Table 1 (*p* < 0.05). Moreover, neither *LM*_*o*_ nor *LM*_*a*_ were statistically significantly related to the flow rate at the falling stage (i.e., water level rate < 0). The mass fluxes at the falling stage are independent of the flow rate because the macro-debris can become trapped on the river bank when the water level drops^42^. In fact, the mass fluxes at the falling stage were non-significantly regressed with the flow rate (*p* > 0.05; see Table 1).

**Table 1:** Model parameters of the mass fluxes.

	Stage	δ_ln (*L*)_^*b^	ln(*a*)^*c^	b^*d^	*n*	*r*^*2*^	*p*
*LM*_*o*_	Rising	1.6	−101 ± 25	18 ± 4	16	0.54	1.1 × 10^−3^
	Falling	2	9 ± 18	−2 ± 3	13	0.043	5.0 × 10^−1^
*LM*_a_^*a^	Rising	1.3	−40 ± 20	8 ± 4	16	0.25	4.7 × 10^−2^
	Falling	2	−20 ± 20	3 ± 3	13	0.079	3.5 × 10^−1^

^*a^The mass flux was estimated by multiplying *LA*_o_ by the mean M/A.

^*b^The error of the mass flux (δ_ln(*L*)_) is defined by Eq. (6).

^*c^The error of ln (*a*) (δ_ln(*a*)_) is defined by Eq. (3).

^*d^The error of *b* (δ_*b*_) is defined by Eq. (4).

The mass fluxes were estimated by applying the *L-Q* equations for *LM*_*o*_ and *LM*_*a*_ to the flow rates at the rising stage, which are referred to as *LM*_*e-o*_ and *LM*_*e-a*_, respectively. Both *LM*_*e-o*_ and *LM*_*e-a*_ were significantly correlated with *LM*_*o*_ (Fig. 3c; *n* = 16, *r* = 0.757, and *p* < 0.001 for *LM*_*e-o*_; *n* = 16, *r* = 0.831, and *p* < 0.001 for *LM*_*e-a*_). In addition, the RMSE of *LM*_*e-a*_ (1.6 g/s/m) was slightly smaller than the RMSE of *LM*_*e-o*_ (4 g/s/m). The uncertainty in the mass flux estimation is discussed below. Nevertheless, the estimation error of *LM*_*e-o*_ was equivalent to that of *LM*_*e-a*_; hence, this result demonstrates that the area fluxes calculated by our algorithm are useful for understanding the relationship between the mass fluxes and flow rate.

## Discussion

Our algorithm enables us to safely and efficiently measure the area flux and transport velocity of the floating macro-debris on a river surface without debris sampling. To date, riverine macro-debris has been less monitored, although this necessity has rapidly increased according to the progress of plastic pollution in the world’s oceans because rivers are major sources of plastic debris. Monitoring riverine debris is essential to reduce the emissions from land to the ocean and to obtain scientific data related to the transport of floating riverine macro-debris. Nevertheless, there are three main issues in evaluating the mass fluxes by river video monitoring: (1) evaluation of the mass fluxes, (2) conversion of the plastics mass fluxes from the total mass fluxes, and (3) vertical distribution of the mass fluxes.

**Figure 4:**
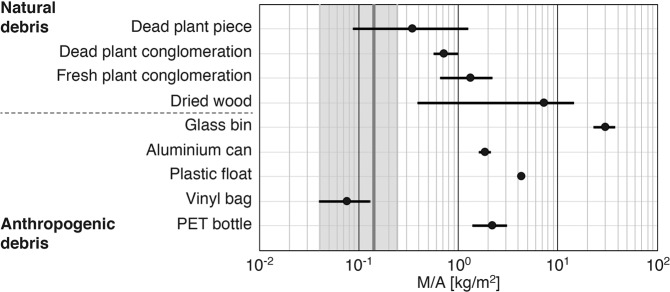
M/A of the various types of potential floating macro-debris. The black bar is the range between the maximum and minimum M/A values, and the black circle is its mean. The light grey area is the 95% confidence interval of the M/A value of the macro-debris collected from the Edo River, and the dark grey line is its mean (0.14 kg/m^2^).

To evaluate the mass flux determined via river video monitoring, the M/A value of the macro-debris floating on the surface of a target river was needed. In the Edo River, the mean M/A value of the debris was 0.14 kg/m^2^ (dark grey line in Fig. 4) and had a 95% confidence interval ranging from 0.040 to 0.24 kg/m^2^ (light grey area in Fig. 4). To examine the M/A value of the debris in the Edo River, we investigated the M/A values of various potential macro-debris used in the laboratory experiment. In addition, the M/A values of twenty dead plant pieces from dead plant conglomerations (items 18–20) were also measured. The M/A values of polyethylene terephthalate (PET) bottles, plastic flotation devices, aluminium cans, and glass vessels were on the order of 1, 1, 1, and 10 kg/m^2^, respectively, while the M/A value of vinyl bags had a wider range compared with the M/A values of the other anthropogenic debris because the surface area of the vinyl bags varied greatly according to the flowing state, such as a crumpled or bent state (Fig. 4). The M/A of natural debris had a wide range compared with that of the anthropogenic debris. In particular, both M/A values of the dead plant pieces and dried wood greatly varied on the order of 0.1 and 10 kg/m^2^. Hence, M/A depends strongly on the composition of the floating macro-debris in target rivers. In the Edo River, natural debris dominates the total debris (Supplementary Fig. S3), and its mass percentage was 69–100%. Thus, the mean M/A value ranged between 0.01 and 0.1 kg/m^2^. The mass percentage and M/A values of the floating macro-debris collected in target rivers, therefore, should be measured; then, the M/A value of the total debris can be estimated by summarizing the M/A values weighted by the mass percentages.

Quantifying the floating riverine macro-plastics is essential to take countermeasures against plastic emissions into the oceans. A simple solution for quantifying floating macro-plastics is to calculate its mass flux by multiplying the overall mass flux by the mass percentage of plastics. In the present study, the mass percentage of anthropogenic debris (not only plastics) ranged from 0% to 31% by weight. Its mean was 4.2 ± 1.6% (SEM). Additionally, Gasperi *et al*.^21^ demonstrated that the mass percentage varied between 0.8% and 5.1%, and the highest plastics mass percentage was obtained by the floating debris-retention booms installed near the outlet of the largest combined sewer outflows within the Paris metropolitan area^43^. This observation indicates that the mass percentage reflects the land use in the river basin. Hence, at present, to calculate the mass plastics fluxes from the total mass fluxes, we need to measure the mass percentage in target rivers, e.g., using booms.

Our algorithm might also be useful in measuring the mass flux of floating macro-plastics. The amount of macro-plastics on beaches has been sequentially monitored by applying image analysis focused on the colour of the plastics in photographs taken by webcams^26,28,44^. Because the colour of plastics differs greatly from the colour of natural debris, the characteristic plastic colour might resolve this issue of determining the mass flux of plastics. In fact, as shown in Fig. 1c, g, the colour difference of plastics from the river surface was larger than that of the natural debris. In the experiment classifying floating macro-debris into natural and anthropogenic debris by the colour difference (e.g., >80: anthropogenic debris) using the 29 videos of the Edo River, the area percentage of anthropogenic debris ranged from 0% to 44%, and its mean was 4.5 ± 1.5% (SEM), consistent with the mean mass percentage (i.e., 4.2 ± 1.6%). However, it is difficult to statistically compare the mass and area percentages because the mass percentages were very low in the 29 observations (Supplementary Table S2). Meanwhile, to quantify the transport of floating macro-plastics, we need to develop a detection technique of plastics in the future. The detection of plastics can be improved using cameras extended beyond the visible band^37,45–48^. Recently, several researchers have studied the application of near-infrared hyperspectral cameras to identify plastics in the environment^45–48^. If hyperspectral imaging is available in rivers, macro-plastics can be distinguished from the macro-debris flowing on the river surface. Because hyperspectral imaging can identify polymer materials, the mass fluxes of macro-plastics can be calculated from the area fluxes using the M/A value of each polymer material and/or item. Thus, the use of a hyperspectral camera might also resolve the issues of the difference between the area and mass percentages. A combination of our algorithm and hyperspectral imaging, therefore, could be a useful tool to measure the mass fluxes of macro-plastics in the future.

Meanwhile, when quantifying the mass flux of macro-plastics using our algorithm, the uncertainty of the mass flux would propagate due to several parameters, such as the M/A, coefficients of Eq. (1) (i.e., *a* and *b*) and mass percentage of macro-plastics. In the quantification of the mass flux, the error of M/A initially propagates to the error of *LM*_*a*_, subsequently to the error of the coefficients of Eq. (1) (*a* and *b*), and finally to the mass flux estimation of macro-plastics. From the mass flux calculation ($L{M}_{a}=(M/A)\times L{A}_{o}$), the error of *LM*_*a*_ (${\delta }_{L{M}_{a}}$) is estimated using the following equation:

2 $${\delta }_{L{M}_{a}}={\delta }_{M/A}\times L{A}_{o}$$

where ${\delta }_{M/A}$ is SEM of M/A, namely, 0.05 kg/m^2^. By substituting the mean of *LA*_*o*_ (0.022 m^2^/s/m; see Supplementary Table S2) into Eq. (2), ${\delta }_{L{M}_{a}}\,$was 1.1 g/s/m smaller than the RMSE between *LM*_*o*_ and *LM*_*a*_ (4 g/s/m). Meanwhile, the error of *LM*_*a*_ would propagate to the coefficients (*a* and *b*) of Eq. (1). From the linearization of Eq. (1), the uncertainties of *a* and *b* are estimated as follows:

3 $${\delta }_{\mathrm{ln}(a)}={\delta }_{\mathrm{ln}(L)}\sqrt{{\sum }^{}{(\mathrm{ln}(Q))}^{2}/\Delta }$$

4 $${\delta }_{b}={\delta }_{\mathrm{ln}(L)}\sqrt{N/\Delta }$$

5 $$\Delta =N{\sum }^{}{(\mathrm{ln}(Q))}^{2}-{(\sum \mathrm{ln}(Q))}^{2}$$

where *N* is the number of data points used to determine *a* and *b*. The lower and upper bounds of the summation are 1 and *N*, respectively; ${\delta }_{\mathrm{ln}(a)}$ and ${\delta }_{b}$ represent the errors of *a* and *b*, respectively. The uncertainty of *LM*_*a*_ (${\delta }_{\mathrm{ln}(L)}$) is estimated using the following equation:

6 $${\delta }_{\mathrm{ln}(L)}=\sqrt{\frac{1}{N-2}{\sum }^{}{(\mathrm{ln}(L{M}_{o})-\mathrm{ln}(a)-b\mathrm{ln}(Q))}^{2}}$$

where $\mathrm{ln}(L{M}_{o})$ and $\mathrm{ln}(Q)$ are the natural logarithms of *LM*_*o*_ and *Q*, respectively. Consequently, the uncertainties of ${\delta }_{\mathrm{ln}a}$ and ${\delta }_{b}$ are shown in Table 1. The uncertainties of *a* and *b* for *LM*_*a*_ were equivalent to those for *LM*_*o*_. On the other hand, the SEM of the mass percentage was 1.6%, and hence 38% (=1.6%/4.2%) of its relative error would propagate to the mass flux estimation of macro-plastics. Nevertheless, we postulate that our algorithm based on the river video monitoring data plays a role as a substitute tool for debris collection from the river surface because the RMSE of *LM*_*e-a*_ (1.6 g/s/m) was slightly smaller than *LM*_*e-a*_ (4 g/s/m).

Furthermore, macro-debris could be transported via river water with a vertical distribution due to turbulent flow. The macro-debris flowing below the river surface cannot be measured by our algorithm. This condition is a limitation of river surface monitoring. One approach to resolving this issue is to consider a vertical distribution model according to the type of macro-debris. To date, vertical distribution models of various materials, such as sediments^49,50^, nutrients^51^, soil organic carbon^52^ and plastic debris^42^, have been investigated in rivers. These consider the influences of water flow, wind, vertical mixing, and buoyance/settling depending on the specific gravity of the materials. The goal of this study was to evaluate the mass fluxes of macro-plastics, which are secondary sources of micro-plastics. The total mass fluxes of macro-plastics in river cross-sections will be evaluated by considering a vertical distribution model in the future^42^.

In the present study, an algorithm for quantifying the transport of floating macro-debris by river video monitoring has been described. Applying our algorithm would be helpful to more easily and safely establish the relationship of the mass fluxes and flow rates compared with the collection of floating macro-debris, although several field surveys will be needed to accurately estimate the mass fluxes. The establishment of this relationship permits us to estimate the mass flux from the flow rate observed at each station. In particular, quantifying the mass flux at the rising stage is essential to evaluate the emission of floating macro-debris from rivers because the mass fluxes at the rising stages were found to be one to two orders of magnitude greater than those at the falling stages/under normal flow conditions (Supplementary Table S2). In the future, we will quantify the floating macro-debris in rivers by applying our algorithm to videos recorded under various flow conditions.

## Conclusions

Floating macro-debris transport can be successfully quantified by monitoring the river surface using a digital video camera and applying an image processing technique based on the colour difference of the floating macro-debris. Our algorithm can capture the area flux of the debris, which is its covered area per unit time. The area flux (*LA*_*o*_) can be converted into the mass flux (*LM*_*a*_) using the mass per unit area (M/A) of the floating macro-debris. *LM*_*a*_ was significantly regressed with the flow rate in the river at the rising stage and was consistent with the mass flux (*LM*_*o*_) measured by collecting floating macro-debris. If the mass percentage of macro-plastics to the total debris in a river is obtained, the mass flux of floating macro-plastics can be quantified. Quantifying the floating macro-plastics in rivers is essential to formulate countermeasures and mitigate the adverse impacts of land-based loads, such as plastic pollution. Our algorithm could be the basis for understanding the transport of floating riverine macro-plastics around the world and consequently can contribute to more effective countermeasures.

## Methods

### Fundamental technique for generating difference images from video data

**Figure 5:**
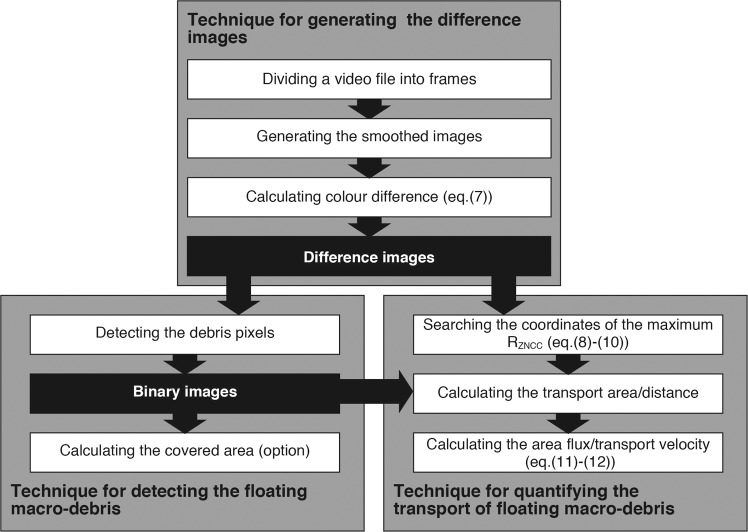
Flowchart for quantifying the transport of floating riverine macro-debris.

The fundamental technique for generating difference images from video data consists of three steps (Fig. 5): (1) dividing a video file (e.g., m2ts, mp4, and mov) into multiple frame images (e.g., jpeg, and png; Fig. 1a,e, respectively), (2) generating a smoothed image from each frame image (Fig. 1b,f), and (3) computing the colour difference between the original and smoothed images in the CIELuv colour space converted the RGB colour space (Fig. 1c,g).

In the first step, a video file is divided into multiple frame images based on the analysis frame rate. First, the divided frame images are smoothed using a uniform box filter (5 px × 5 px) to remove noise. The divided frame image after smoothing is used as the original frame image. In the present study, the analysis frame rate was arbitrarily selected as 5 frames per second (fps) considering the flow of floating macro-debris in the video. Video data are often obtained at a high filming frame rate (e.g., 29.97 fps). The computational load could be excessive if the analysis frame rate is too high when the video data are divided. Conversely, if the analysis frame rate is too low, we might not correctly detect floating macro-debris.

To effectively extract the pixels of floating macro-debris, the smoothed image is generated using the original frame image (Fig. 1b,f). For smoothing, the median filter is applied to the original frame image, and the window size of the filter is 200 px × 200 px in the present study. The window size is arbitrary, and we determined this window size through trial and error in the present study. If the window size is too small, it is difficult to extract floating macro-debris because the colours in the original frame image are similar to those in the smoothed image.

To quantify the difference in colour between the original and smoothed images, the RGB colour space of both images is converted into the CIELuv colour space (see Supplementary Notes). The CIELuv colour space attempts to obtain a perceptual uniformity of the colour difference in the three-dimensional space (i.e., L^*^, u^*^, and v^*^)^41^. The colour difference *ΔE* is expressed by the Euclidean distance between two points in the CIELuv colour space as follows:

7 $$\varDelta E=\sqrt{{({L}_{1}^{\ast }-{L}_{2}^{\ast })}^{2}+{({u}_{1}^{\ast }-{u}_{2}^{\ast })}^{2}+{({v}_{1}^{\ast }-{v}_{2}^{\ast })}^{2}}$$

where the subscripts of 1 and 2 denote the original and smoothed images, respectively. The colour difference between the original and smoothed images is computed using Eq. (7) (Fig. 1c,g).

### Fundamental technique for detecting the pixels of floating macro-debris from the difference images

The pixels of macro-debris can be extracted by determining the appropriate constant threshold value in the difference image (hereafter referred to as the binary image) (Fig. 1d,h). In the present study, the threshold value is 10 for the laboratory experiments and 20 for the river video monitoring data. Notably, the application of a higher threshold value in river video monitoring is a better approach because the waves generated on the water surface are potentially mis-detected. The area covered by floating macro-debris is computed by multiplying the number of debris pixels in the binary image by the area of a single pixel. For each video, the optimum threshold value must be selected by trial and error compared with the covered area calculated through visual observation (Fig. 2a,c).

### Fundamental technique for measuring the area flux and transport velocity of floating macro-debris using the difference images

The flux and velocity of floating macro-debris are measured using multiple difference images. In the present study, the flux was defined as the area of floating macro-debris transported per unit time and unit width (e.g., m^2^/s/m). In general, the flux is often evaluated as the mass of floating macro-debris (e.g., g/s/m). However, it is difficult to directly evaluate the mass flux from two-dimensional video data because the vertical size and specific gravity of the debris are unknown. First, the area flux is evaluated using our algorithm.

**Figure 6:**
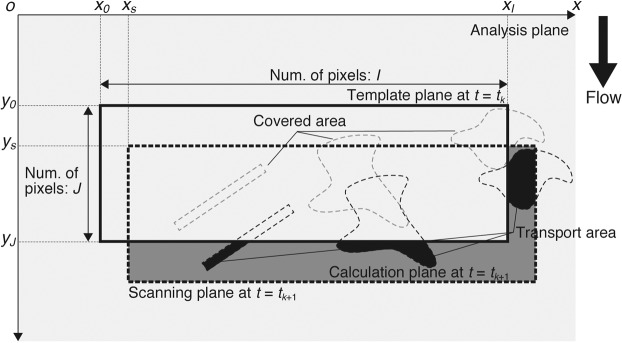
Schematic image of the area flux calculation by applying the template matching method. The template plane (bold outline area) is defined in the analysis plane (light grey area) at *t* = *t*_*k*_, and its position is arbitrary. *I* and *J* are the numbers of pixels in the *x* and *y* directions, respectively. The scanning plane (bold dashed outlined area) is searched in the analysis plane at *t* = *t*_*k*+1_ using the template matching method (Eq. (8)). The differential area between the template and scanning planes is the plane used to calculate the area flux (grey area) and is called as calculation area. The number of debris pixels in the calculation plane, namely, the transport area (black area), is counted. The area flux is calculated with Eq. (11) using the number of debris pixels. Note that the floating macro-debris is shown by the grey and black dashed outline areas at *t* = *t*_*k*_ and *t* = *t*_*k*+1_, respectively.

The area flux and velocity can be computed by applying the template matching method to two difference images at steps *t*_*k*_ and *t*_*k*+1_. Note that the subscript *k* is a time step index. First, as shown in Fig. 6, the template plane is defined from the difference image $\varDelta E$ at step *t*_*k*_ in the analysis plane, where *x* and *y* are the coordinates in the lateral and flow directions, respectively, and $\,({x}_{0},{y}_{0})$ are the origin coordinates of the template plane in the $x-y$ plane. Notably, the size of the template plane should be as large as possible because the precision of template matching method is increased by tracking many objects in the template plane during $\Delta t$ (=*t*_*k*+1_ − *t*_*k*_) corresponding to 1/(analysis frame rate). The template plane is then searched in the difference image at step *t*_*k*+1_ by calculating the zero-mean normalized cross-correlation (ZNCC)^53^ as follows:

8 $${R}_{ZNCC}({x}_{s},{y}_{s},{t}_{k})=\frac{{\sum }_{j=0}^{J-1}{\sum }_{i=0}^{I-1}\{(S({x}_{i}+{x}_{s},{y}_{j}+{y}_{s},{t}_{k+1})-\bar{S})(T({x}_{i},{y}_{j},{t}_{k})-\bar{T})\}}{\sqrt{{\sum }_{j=0}^{J-1}{\sum }_{i=0}^{I-1}{(S({x}_{i}+{x}_{s},{y}_{j}+{y}_{s},{t}_{k+1})-\bar{S})}^{2}{\sum }_{j=0}^{J-1}{\sum }_{i=0}^{I-1}{(T({x}_{i},{y}_{j},{t}_{k})-\bar{T})}^{2}}}$$

9 $$\bar{S}=\frac{1}{IJ}\mathop{\sum }\limits_{j=0}^{J-1}\mathop{\sum }\limits_{i=0}^{I-1}S({x}_{i}+{x}_{s},{y}_{j}+{y}_{s},{t}_{k+1})$$

10 $$\bar{T}=\frac{1}{IJ}\mathop{\sum }\limits_{j=0}^{J-1}\mathop{\sum }\limits_{i=0}^{I-1}T({x}_{i},{y}_{j},{t}_{k})$$

where *x*_*i*_ and *y*_*j*_ are the lateral and flow positions at pixel (*i*, *j*), and $T$ is $\varDelta E$ at position $({x}_{i},{y}_{j})$ and step *t*_*k*_ in the template plane. In addition, $S$ is $\varDelta E$ at position $({x}_{i}+{x}_{s},{y}_{j}+{y}_{s})$ and step *t*_*k*+1_ in the scanning plane, and $({x}_{s},{y}_{s})$ are the origin coordinates of the scanning plane. Note that the size of the scanning plane is the same as that of the template plane. As shown in Eqs. (9) and (10), $\bar{S}$ and $\bar{T}$ are the spatial averages of the pixel values in the scanning and template planes, respectively. $I$ and $J$ are the maxima of the pixels in the *x* and *y* directions of the template plane, respectively. For the laboratory channel and river, the analysis and template planes are shown in Fig. 1c,g, respectively.

The area flux *LA*_*o*_ is calculated by the following equations:

11 $$L{A}_{o}=\mathop{\sum }\limits_{j=0}^{J-1}\mathop{\sum }\limits_{i=0}^{I-1}rC({x}_{i}+{x}_{smax},{y}_{j}+{y}_{smax},{t}_{k+1})/\Delta t$$

12 $$\{\begin{array}{c}u=({x}_{smax}-{x}_{0})/\Delta t\\ v=({y}_{smax}-{y}_{0})/\Delta t\end{array}$$

where $C$ is the pixel value in the calculation plane of the binary images, which is the portion of the scanning plane except the range that overlaps the template plane at step *t*_*k*_ (Fig. 6), and $r$ is the area of a pixel. Note that the calculation plane is defined in the binary image after detecting the debris pixels (see Fig. 5). When ${R}_{ZNCC}$ is maximized, $({x}_{s},{y}_{s})$ becomes $({x}_{smax},{y}_{smax})$. In addition, $u$ and $v$ are the transport velocities in the lateral and flow directions, respectively. In the present study, the lateral transport in the *x*-axis was disregarded because the *y*-axis in the frames is almost parallel to the flow direction. Finally, the area fluxes in all frames are averaged over the filming time.

### Laboratory experiment in the open channel

To evaluate the performance of our algorithm, we conducted a laboratory experiment by filming twenty natural and anthropogenic items (see Supplementary Notes and Table S1) floating on the water surface of the open channel in our laboratory on 6 May and 13 September 2019. The open channel is 1.0 m wide, 1.8 m high, and 20 m long (Supplementary Fig. S4). A pump and flow meter were installed upstream of the open channel; thus, the flow rate could be adjusted in the range of 0.0001 m^3^/s to 0.2535 m^3^/s. In this experiment, the flow rate was 0.055 m^3^/s, and thus, the uniform water depth was set to 0.58 m. Videos were recorded perpendicularly using a video camera (HDR-XR550V; Sony, Japan) fixed at the lateral centre on the upper edge of the channel. The filming speed or frame rate was 29.97 fps, and the camera resolution was 1920 × 1080.

### Collection and video monitoring of the floating macro-debris in the Edo River

The collection and video monitoring of floating macro-debris were performed at the Noda Bridge across the Edo River (Supplementary Fig. S5) during two typhoon events in 2010 (Supplementary Notes) to verify the *in situ* applicability of our algorithm.

Floating macro-debris was collected using the collection equipment for 1–5 min (see Supplementary Table S2). Simultaneously, a video of the river surface was perpendicularly recorded from the bridge using a video camera (HDR-XR550V; Sony, Japan). The video frame rate was 29.97 fps, and the camera resolution was 1920 × 1080. The collection equipment was included in the filmed video footage. The spatial range of the video camera was approximately 3.0 m in the flow direction and 4.0 m in the lateral direction. Because the location of the collection equipment in the river surface changed horizontally and vertically according to the flow conditions during recording, the horizontal location was captured (see Supplementary Notes). Additionally, the spatial resolution changed depending on the vertical location and water level, and the mean spatial resolution recognizable as debris was 8.0 cm^2^/px within a range of 5.9–14 cm^2^/px (see Supplementary Notes), corresponding to the size of macro-debris (>2.5 cm^10^). By analysing the area upstream of the collection equipment in the video (see Fig. 1e), we can compare the area flux calculated via video monitoring with the mass flux measured by debris collection.

### Statistical analysis

Correlation and regression analyses were conducted using R version 3.3.2 (2016–10–31). In the correlation analysis, the Pearson correlation coefficient was calculated and evaluated to identify any statistically significant relationships between the mass and area fluxes and between the observed and estimated mass fluxes. Moreover, regression analysis was conducted to test the statistical significance of the relationship between the mass flux and flow rate. Both statistical analyses were evaluated at the 95% confidence level.

## Supplementary information

Supplementary Information.

Supplementary Information2.

Supplementary Information3.
