# Supplementary material for: Quantification of floating riverine macro-debris transport using an image processing approach
Source: Sci Rep. 2020 Feb 10;10:2198. doi: 10.1038/s41598-020-59201-1 (PMC7010822; doi:10.1038/s41598-020-59201-1)
Supplement: Supplementary file 1 — Supplementary Information. [file 41598_2020_59201_MOESM1_ESM.pdf]

Quantification of floating riverine macro-debris transport using an image processing approach

Tomoya Kataoka[1]* and Yasuo Nihei[1]

[1] Department of Civil Engineering, Faculty of Science and Technology, Tokyo University of Science, Chiba, 278-8510, Japan

* tkata@rs.tus.ac.jp

Supplementary materials

Supplementary Notes

Supplementary Figure S1. Pictures of the floating macro-debris used in the laboratory experiments.

The covered areas, excluding items 3-5 and 14-20, were calculated by referencing the mesh size of the checkerboard (2 cm or 3 cm) in these pictures. Item 17 in Table S1 was combined with items 14-16. The covered areas in items 14-16 were calculated from the video showing these items flowing along the open channel in the laboratory.

Supplementary Figure S2. Comparison between the original and binary images.

In each panel, the upper and lower snapshots are the original and binary images, respectively. In the lower image, the black pixels indicate macro-debris.

Supplementary Figure S3. Representative image of the floating macro-debris collected from the surface of the Edo River on November 1, 2010.

Supplementary Figure S4. Side view of the open channel in our laboratory.

The open channel is 1.0 m wide, 1.8 m high, and 20 m long. The floating macro-debris flowed along the centre in the lateral direction. The snapshot of the vertical view obtained with the video camera is shown in Fig. 1a.

Supplementary Figure S5. Location of the floating macro-debris collection site.

The black circle is the location of the Noda Bridge across the Edo River.

Supplementary Table S1. Floating macro-debris used in the laboratory experiment.

Supplementary Table S2. Collection and results of the proposed algorithm for the river experiment in the Edo River.

Supplementary Notes

Items of floating macro-debris used in the laboratory experiment

Twenty floating macro-debris items were used in the laboratory experiment, as shown in Supplementary Fig. S1: two polyethylene terephthalate (PET) bottles, three vinyl bags, a plastic flotation device, two aluminium cans, two glass vessels, three wood pieces, four fresh plant conglomerations, and three dead plant conglomerations. Items 14, 15 and 16 are combined into item 17 (not shown in Supplementary Fig. S1). Each item flowed individually along the channel. The weights of all items were measured before the experiment (Supplementary Table S1). The areas covered by the items, excluding the vinyl bags and plant conglomerations, were measured by analysing the pictures of the items (see Supplementary Fig. S1), which were recorded with a digital camera (COOLPIX W300; Nikon, Japan), using image-processing software (ImageJ, downloaded from http://imagej.nih.gov). In addition, the areas covered by the vinyl bags and plant conglomerations were measured using frame images of the videos when these items were floating along the open channel because they exhibited a different flotation behaviour, depending on their floating state.

Collection of floating macro-debris in the Edo River under storm conditions

The macro-debris floating on the river surface was collected at the Noda Bridge (39 km upstream from the river mouth) spanning the Edo River (400 m width, 60 km length, 200 km^2 basin area), which is a branch of the Tone River, the largest river in Japan (Supplementary Fig. S5), on September 24 and from October 31– November 2, 2010. On average, the normal flow rate (185th daily water level in descending order) has been 68

m^3/s for 56 years (1955 to 2010). In the survey periods, the flow rate at the Noda water-level station near the bridge increased because Typhoons Malakas and Chaba approached the south of Japan from September 24-25 and October 29–31, 2010, respectively. Due to the approach of Typhoons Malakas and Chaba, the peak flow rates were 282 m^3/s at 13:30 on September 24 and 455 m^3/s at 3:30 on November 2, 2010, respectively (Supplementary Table S2). The magnitude of the flooding caused by Typhoon Chaba was greater than the flooding caused by Typhoon Malakas.

Floating macro-debris were collected by hanging a 2.5-cm mesh net that was secured within a 1.0-m wide and 1.0-m high (external) frame of steel pipes. A water gauge (HOBO U20-001-04, Onset Computer Corporation, USA) was attached at the bottom edge of the frame because the vertical position of the net from the water surface was recorded. The net was hung from the bridge at the centre of the stream for 1-5 min, according to how full the net became. The floating macro-debris were classified into natural and anthropogenic debris, and the mass of each type was measured after drying the material in a drying furnace at 80 °C. The mass flux of the floating macro-debris was calculated by dividing the dried total mass by the time the net hung from the bridge.

Identification of the location of the equipment used to collect the floating macro-debris

When collecting floating macro-debris at the Noda Bridge over the Edo River, the collection equipment swung across the river surface, and its location always changed during collection according to the river flow (Fig. 1e). The equipment location must be identified in each frame to evaluate the area fluxes of the debris collected by the equipment. In the identification of the equipment location, the characteristics of the net

colour were used in the HSV colour space because of the efficiency of identification. In the HSV colour space, a colour is expressed by its hue, saturation, and value[1]. The hue is the type of colour (e.g., red, blue, or green), and it is expressed as an angle ranging from 0° to 360°. Notably, 0° is the same as 360°. The saturation is the colour chroma and is expressed as a percentage ranging from 0 to 100%. The value is the lightness of a colour and is expressed as a percentage ranging from 0 to 100%.

In the first step used to identify the equipment location, the colour space was converted from the RGB colour space to the HSV colour space because the pixel colours of the video are usually expressed in the RGB colour space, where the degrees of red, green, and blue are each expressed as numbers ranging from 0 to 255. The equipment location was captured by searching the pixels in the ranges of $45^\circ < \text{hue} < 85^\circ$ and $\text{saturation} > 45\%$ in the HSV colour space. For the conversion of the colour space, we used the function "cvtColor" in the OpenCV 3.4.1 program. The coordinates of the left and right edges of the equipment were identified. The difference between both edges corresponds to the width of the equipment. When calculating the area fluxes, the debris pixels were counted in the lateral range of the equipment width.

Spatial resolution of floating macro-debris that is recognizable using video monitoring

The spatial resolution at which floating macro-debris is recognized in video monitoring depends on the distance from the video camera to the water surface. In the laboratory experiments, the spatial resolution was defined by dividing the channel width (100 cm) by the number of pixels of the water surface in the lateral direction (1340 pixels), namely, $(100/1340)^2$ cm^2/px. Meanwhile, in the river experiment, the spatial resolution

changes according to the variability of the river water level and the vertical location of the collection equipment. In the present study, the 1-m frame of the collection equipment was used as a reference to determine the spatial resolution. The width of the collection equipment captured in each video using the method described above varied in the range of 269 px to 413 px among the 29 videos. The images in the original frames were initially smoothed with a uniform box filter (5 px × 5 px) to remove any noise in the original images and to detect the debris pixels using our technique. Furthermore, we only analysed conglomerations of pixels greater than 4 pixels to prevent mis-detection. Hence, the mean spatial resolution recognizable as debris was 8.0 cm^2/px within a range of 5.9 cm^2/px (i.e., $4 \times (5 \times 100\ cm/413\ px)^2$) to 14 cm^2/px (i.e., $4 \times (5 \times 100\ cm/269\ px)^2$), corresponding to the size of macro-debris [2] (> 2.5 cm).

Conversion of the RGB colour space into the CIELuv colour space

In the present study, the International Commission on Illumination 1976 (L^* u^* v^*) colour space (CIELuv colour space)[1] was used to define the difference in colour between the debris and background. Notably, L^*, u^*, and v^* are the lightness and red-green and yellow-blue chroma, respectively. Often, the pixels in images have specific red (R), green (G) and blue (B) values in the RGB colour space. The CIELuv values represent one of the colour appearance models recommended by the Commission Internationale de l'Éclairage (CIE) and were calculated from the values of red (R), green (G), and blue (B) in the RGB colour space as follows[3]:

$$L^* = 116(Y/Y_n)^{1/3} - 16 \quad \text{(S1)}$$

$$u^* = 13L^*(u' - u'_n) \quad \text{(S2)}$$

$$v^* = 13L^*(v' - v'_n) \quad \text{(S3)}$$

where u' and v' denote the chromaticity coordinates and are calculated using the following equations:

$$u' = \frac{4X}{X+15Y+3Z} \quad \text{(S4)}$$

$$v' = \frac{9X}{X+15Y+3Z} \quad \text{(S5)}$$

X, Y, and Z in these equations are defined as follows:

$$\begin{pmatrix} X \\ Y \\ Z \end{pmatrix} = \begin{pmatrix} 0.412453 & 0.357580 & 0.180423 \\ 0.212671 & 0.715160 & 0.072169 \\ 0.019334 & 0.119193 & 0.950227 \end{pmatrix} \begin{pmatrix} R \\ G \\ B \end{pmatrix} \quad \text{(S6)}$$

In equations (S2) and (S3), u'_n and v'_n denote the chromaticity coordinates indicated as white. For the conversion of the colour space, we used the function cvtColor in the OpenCV 3.4.1 program.

References

1. Fairchild, M. D. Color Appearance Models (Second edition). 385 (John Wiley & Sons Ltd, 2013).

2. Kershaw, P. J., Rochman, C. M. Sources, Fate and Effects of Microplastics in the Marine Environment: A Global Assessment. *Rep. Stud.* **90**, 97 (2016).

3. Kako, S. i., Isobe, A. & Magome, S. Low altitude remote-sensing method to monitor marine and beach litter of various colors using a balloon equipped with a digital camera. *Mar. Pollut. Bull.* **64**, 1156-1162 (2012).

Supplementary Figure S1 **Kataoka and Nihei**

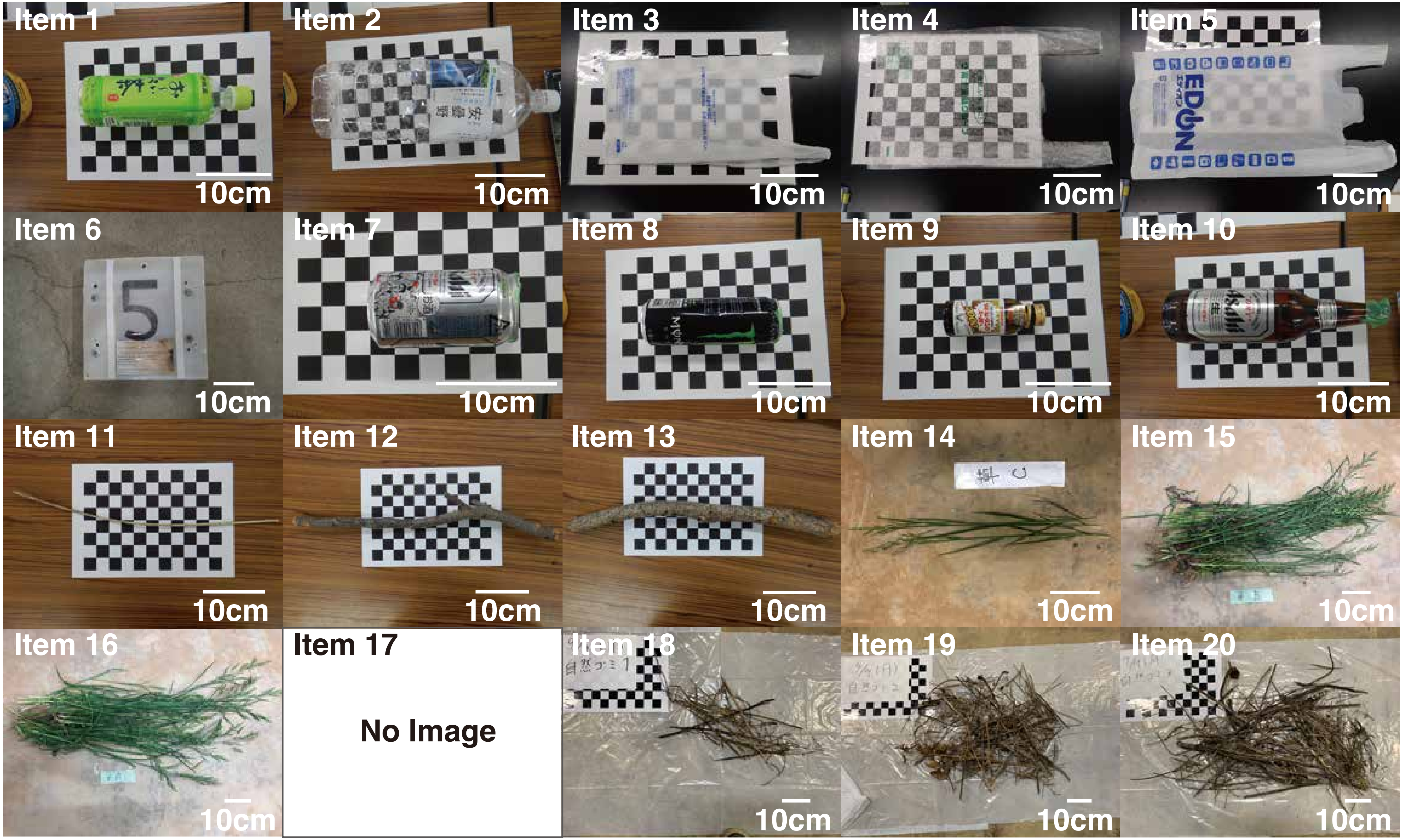

Supplementary Figure S1. Pictures of th1 floating macro-debris used in the laboratory experiments.
The covered areas, excluding items 3-5 and 14-20, were calculated by referencing the mesh size of the checkerboard (2 cm or 3 cm) in these pictures. Item 17 in Table S1 was combined with items 14-16. The covered areas in items 14-16 were calculated from the video showing these items flowing along the open channel in the laboratory.

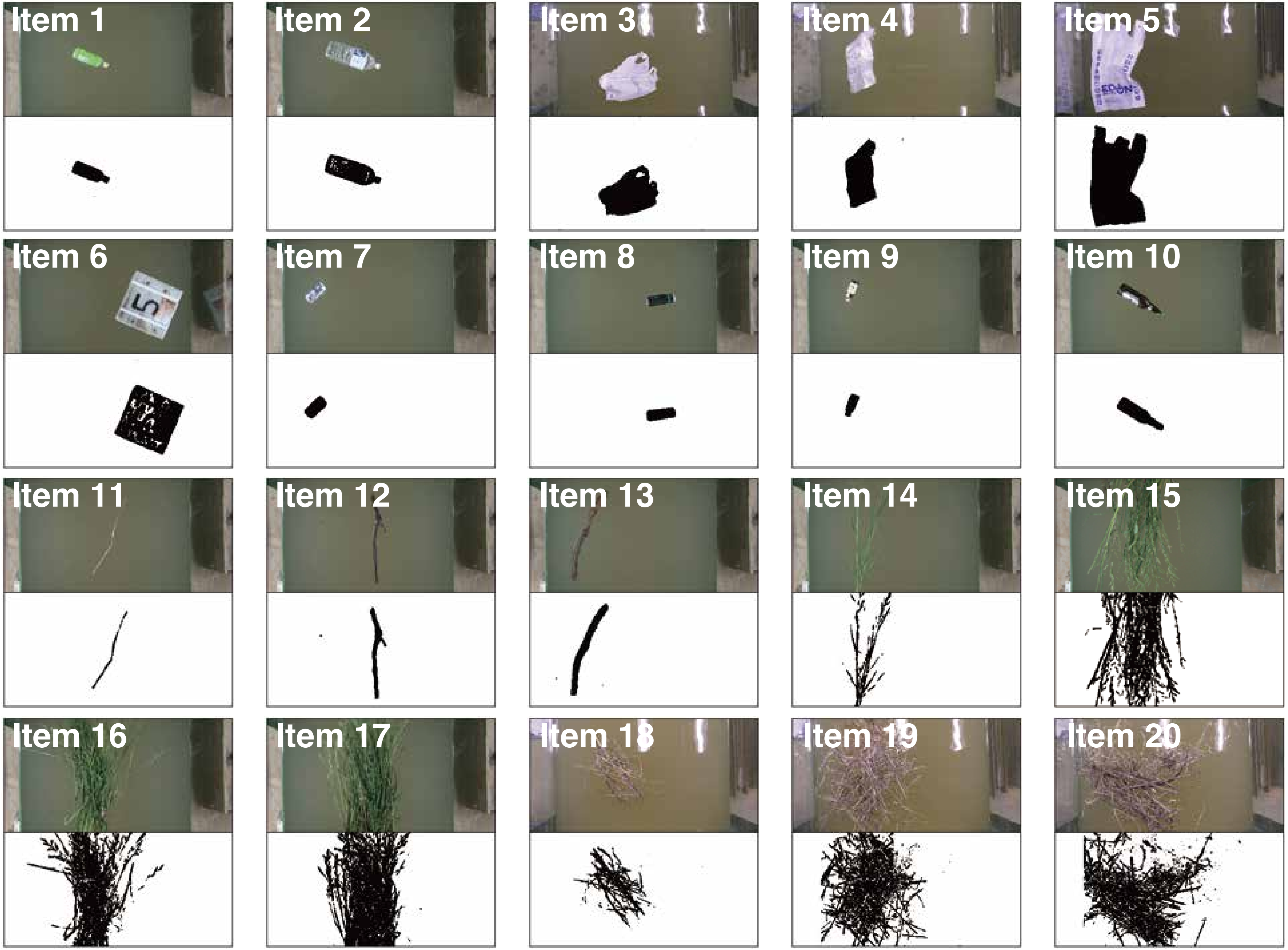

Supplementary Figure S2. Comparison between the original and binary images.
In each panel, the upper and lower snapshots are the original and binary images, respectively. In the lower image, the black pixels indicate macro-debris.

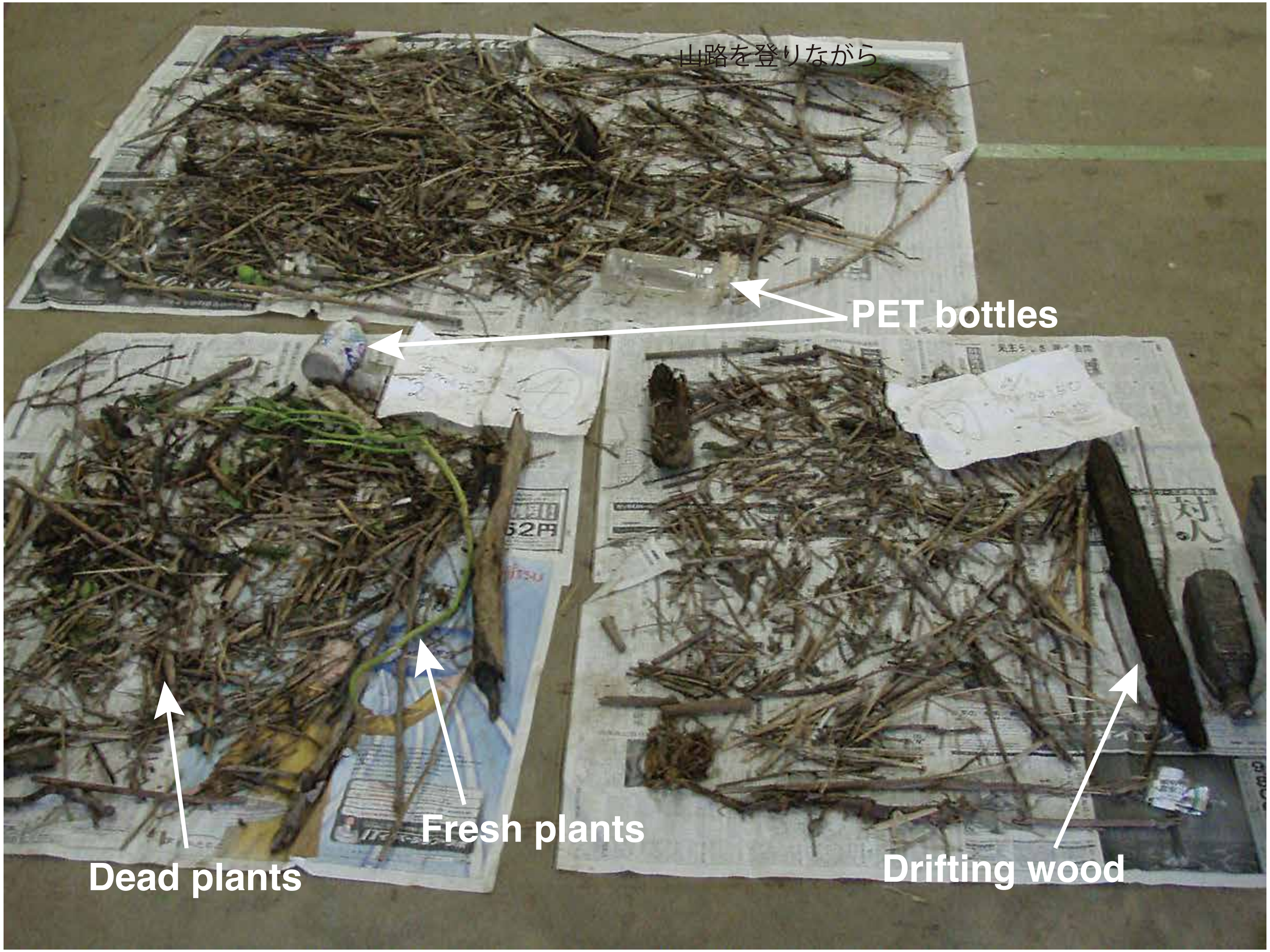

Supplementary Figure S3. Representative image of the floating macro-debris collected from the surface of the Edo River on November 1, 2010.

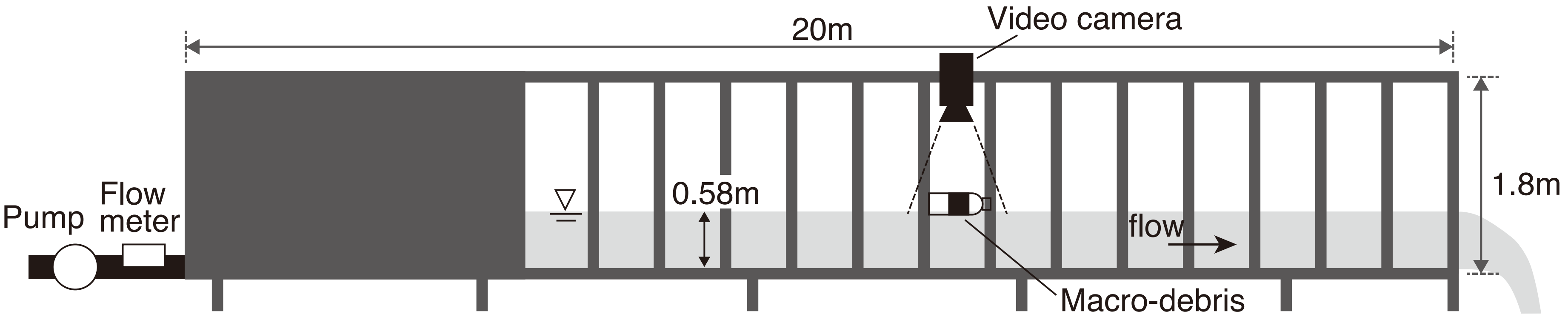

Supplementary Figure S4. Side view of the open channel in our laboratory.
The open channel is 1.0 m wide, 1.8 m high, and 20 m long. The floating macro-debris flowed along the centre in the lateral direction. The snapshot of the vertical view obtained with the video camera is shown in Fig. 1a.

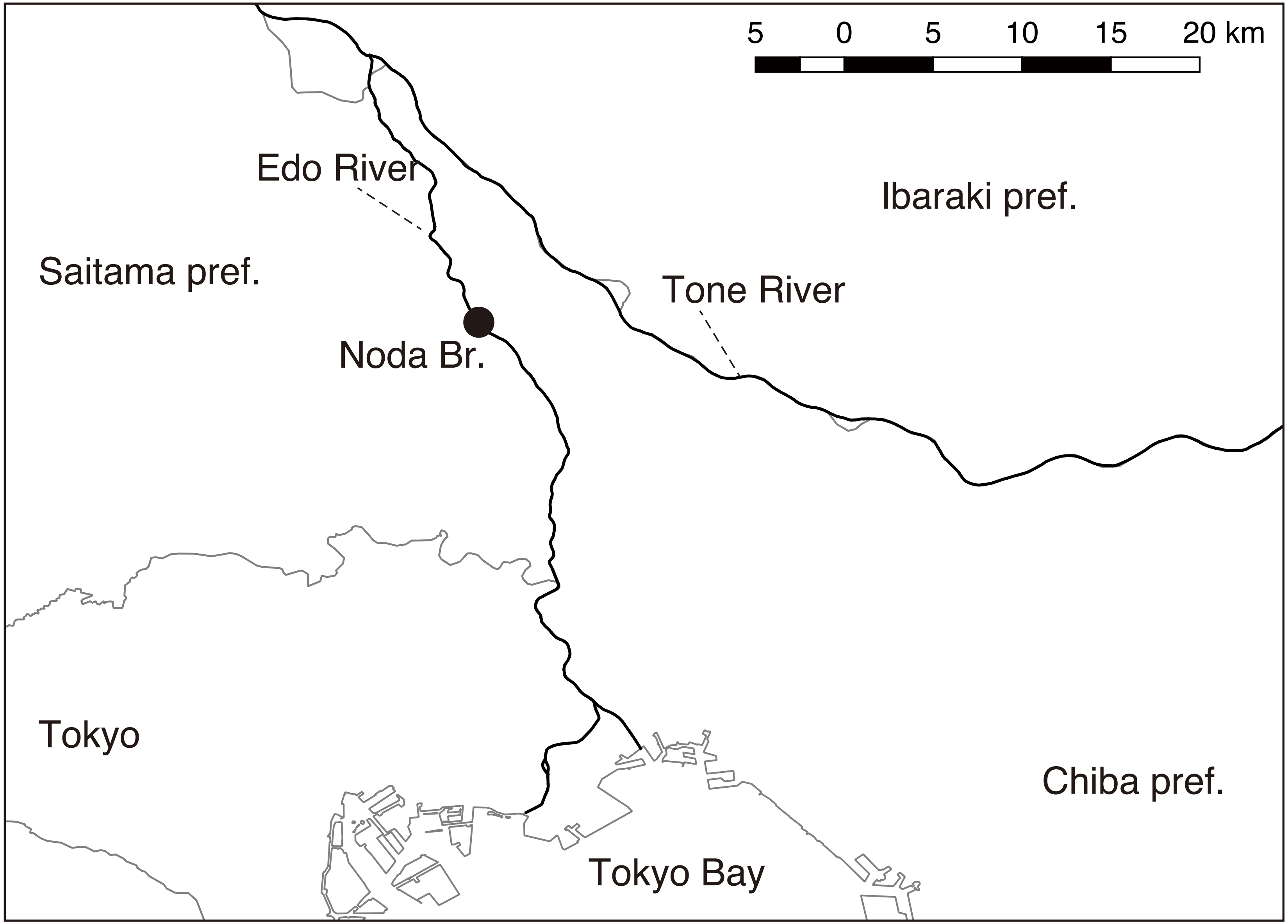

Supplementary Figure S5. Location of the floating riverine macro-debris collection site.
The black circle is the location of the Noda Bridge across the Edo River.
